# Pre-COVID health-related quality of life predicts symptoms and outcomes for patients with long COVID

**DOI:** 10.3389/fpubh.2025.1581288

**Published:** 2025-07-11

**Authors:** Brittany Lapin, Samantha Baker, Nicolas Thompson, Yadi Li, Alex Milinovich, William Lago, Irene Katzan

**Affiliations:** ^1^Department of Quantitative Health Sciences, Lerner Research, Cleveland Clinic, Cleveland, OH, United States; ^2^Neurological Institute Center for Outcomes Research and Evaluation, Cleveland Clinic, Cleveland, OH, United States; ^3^Primary Care Institute, Cleveland Clinic, Cleveland, OH, United States

**Keywords:** patient-reported outcomes, health-related quality of life, PROMIS Global Health, long-COVID symptoms, post-acute sequelae of COVID

## Abstract

**Background:**

Post-acute sequelae SARS-CoV-2 (PASC) is a prevalent condition with variable symptom presentation. PASC occurs more often with pre-existing medical conditions, however it is unknown whether pre-COVID health-related quality of life (HRQL) is associated with PASC. Similarly, the trajectory of HRQL following PASC is unknown.

**Objective:**

Our study sought to evaluate (1) whether pre-COVID HRQL is associated with PASC symptoms; (2) whether PASC patients have worse pre-COVID HRQL compared to matched controls; and (3) to compare HRQL trajectories from pre-COVID to 1-year follow-up between PASC patients and matched controls.

**Design:**

Retrospective cohort study with propensity-score matched control group.

**Participants:**

The cohort included 1,114 adult patients (mean age 53 ± 14, 75% female) seen in a PASC clinic between 2/10/21 and 3/27/24 who completed HRQL surveys prior to their initial COVID-diagnosis in a large health system. A propensity-score matched control group included patients with COVID-19 without PASC.

**Main measures:**

HRQL was measured with PROMIS Global Health [global mental health (GMH) and global physical health (GPH) summary scores].

**Key results:**

PASC symptoms were significantly associated with pre-COVID HRQL. Symptoms most associated with PROMIS-GMH included diarrhea/nausea [odds ratio (OR) = 1.27 (95% CI: 1.16–1.39) per five-point worsening] and brain fog [OR = 1.25 (95% CI: 1.14–1.37)], while fatigue [OR = 1.39 (95% CI: 1.15–1.68)] had the highest association with PROMIS-GPH. Pre-COVID GMH and GPH were significantly worse for PASC patients compared to controls [−2.6 (SE 0.4) and −3.4 (0.3) *T*-score points, respectively]. At 1-year following COVID, PASC patients worsened significantly in GMH and GPH (−2.0 ± 8.2 and −1.2 ± 7.5 *T*-score points, respectively), compared to controls who worsened significantly on GMH but not GPH (−0.8 ± 7.7 and 0.2 ± 7.4 *T*-score points, respectively).

**Conclusions:**

In patients with PASC, worse pre-COVID HRQL was associated with more PASC-related symptoms. PASC patients had worse pre-COVID HRQL compared to matched controls and experienced a greater decline in HRQL 1-year after COVID-diagnosis; however, this decline was below the threshold for clinical significance.

## Introduction

An estimated 6%−35% of individuals with COVID-19 have persistent, relapsing, or new symptoms occurring within a few months of initial COVID illness, a condition referred to as post-acute sequelae SARS-CoV-2 infection (PASC) (1–3). This is a heterogeneous condition with variable symptom presentation and severity across individuals, however common symptoms have been reported including post-exertional malaise, fatigue, brain fog, gastrointestinal symptoms, and cough (4–6). Higher risk of developing PASC has been documented for females, older age, higher BMI, and more initial COVID symptoms (7–9). PASC has been found to occur more often in those with pre-existing medical conditions (10–12), including prior sleep problems and fatigue (13), autoimmune disorders, anxiety and depression (14, 15). It has been hypothesized that psychological distress is a risk factor of PASC, with studies showing a history of psychiatric disorders to be an independent predictor of PASC (16). Despite this, it is unknown whether pre-COVID health-related quality of life (HRQL), including mental and physical global health, is associated with PASC.

The trajectory of HRQL following PASC is unclear, with some studies suggesting improvements (17) but most studies indicating HRQL remains affected for up to 2 years (5, 6, 18). However, major limitations of prior research are the lack of baseline, or pre-COVID, HRQL as a reference point. Patient-reported measures of HRQL have been collected routinely at our institution as standard care, providing a unique resource to evaluate change in HRQL of patients with PASC and the effect of pre-COVID HRQL on PASC symptoms and outcomes.

Our study objectives were to evaluate (1) whether pre-COVID HRQL is associated with symptoms of PASC; (2) whether patients with PASC have worse pre-COVID HRQL compared to matched controls; and (3) to compare trajectories of HRQL from pre-COVID to 1-year follow-up between patients with PASC and matched controls.

## Materials and methods

We conducted an observational cohort study of adult patients with PASC seen in a multidisciplinary COVID-19 recovery clinic. The reCOVer Center of Excellence at Cleveland Clinic opened 2/10/21 to care for patients with persistent COVID-19 related symptoms. Patients diagnosed with PASC are referred to the reCOver Clinic for a comprehensive evaluation and, from there, are referred to the appropriate specialty/specialties which have tailored care paths for patients with PASC.

Patients were included in the study if they were ≥18 years of age and visited the reCOVer Clinic between 2/10/2021 and 3/27/2024. For Aims 1 and 2, patients were included in the study if they completed patient-reported outcomes (PROs) as routine care in the year prior to their visit. For Aim 3, patients were included if they also completed PROs at an office visit 1 year following their visit.

The study was approved by Cleveland Clinic's Institutional Review Board (#20-1331). Because the study was minimal risk research involving analyses of pre-existing data, the requirement for patient informed consent was waived.

### COVID control sample

A control cohort of patients with COVID-19 included adults (≥18 years) who tested positive for COVID-19 at Cleveland Clinic as documented in the electronic health record (EHR) as of 2/13/2021. COVID-19 test results, presenting symptoms, and hospitalization outcomes were included from Cleveland Clinic's COVID-19 Registry (19). As the COVID registry now includes millions of patients, a random subset of 12,580 were extracted to serve as potential matched controls. Patients with complete data who had also completed PROs in the year prior to their diagnosis and ~1 year following their positive test result were included as potential controls. While it is possible that some patients with PASC were not referred to the specialized clinic, this is likely uncommon given the clinic's widespread visibility and the consistent referral practices of the providers.

### Electronic health record data

For all patients, COVID-19 clinical outcomes were extracted from the COVID-19 Cleveland Clinic Registry including hospitalization status, intensive care unit (ICU) admission, and symptoms (19). Symptoms were included in the registry based on clinical relevance and extracted from the EHR using natural language processing. At the initial reCOVer Clinic visit, symptoms were evaluated by the clinician and included in the visit note. They were then categorized as never having been experienced, having been experienced prior to COVID infection, resolved following COVID, and a new or recurred symptom following COVID.

### Health-related quality of life

As part of routine care at Cleveland Clinic patients complete questionnaires prior to ambulatory office visits that are tailored to each department. PROMIS Global Health (PROMIS-GH) v 1.0 is captured across most departments in the health system.

PROMIS-GH is a 10-item generic measure of HRQL that evaluates global health and includes a summary score for physical and mental global health (20). PROMIS-GH is standardized to a reference population on a *T*-scale with mean of 50 and standard deviation (SD) of 10 where higher scores indicate better global health. PROMIS-GH has been demonstrated as a valid and reliable measure, with a change of 2.5–5 *T*-score points generally considered a clinically meaningful change (21).

As a measure of pre-COVID global health, all PROMIS-GH measures collected between 1/3/2019 and 3/10/2020 were extracted for patients who were later seen the reCOVer Clinic and controls. For patients with multiple PROMIS-GH measures, the most recent measure was used for analysis.

Additionally, PROMIS-GH was extracted from the EHR for patients who completed it in another clinical department following their reCOVer Clinic visit. These follow-up measures were collected from 6/30/21 through 6/19/24 based on time since the initial visit to the reCOVer Clinic. For controls, follow-up data was pulled through 7/29/24. For patients who completed PROMIS-GH more than once in the follow-up window, the patient's closest survey to 1 year following the reCOVer Clinic visit was included in the analysis. For controls, the first positive COVID test was defined as baseline.

### Statistical analysis

Demographics, comorbidities, initial COVID symptoms and outcomes, and pre-COVID HRQL were summarized for patients with PASC using descriptive statistics. To evaluate for selection bias, characteristics of those who had completed PROMIS-GH and were included in the study were compared to all patients seen in the reCOVer Clinic.

In Aim 1, we evaluated whether pre-COVID HRQL was associated with PASC symptoms. Symptoms were grouped according to whether they were: (1) unresolved symptoms or symptoms that were new following COVID infection (i.e., PASC symptoms); (2) symptoms that were present prior to COVID or resolved following COVID (i.e., Historical symptoms); or (3) symptoms that were never experienced prior to or following COVID (i.e., Never experienced symptoms). Symptoms were evaluated in separate multivariable multinomial logistic regression models, where the primary explanatory variable was PROMIS global physical health (GPH) in Model I and PROMIS global mental health (GMH) in Model II. Models were adjusted for characteristics determined *a priori* as possible confounders: age, sex, and hospitalization for initial COVID infection.

In Aims 2–3, patients with PASC were compared to matched controls. Propensity scores for the probability of having PASC were estimated with a multivariable logistic regression model including the following variables: age, sex, race, BMI, comorbidities (asthma, hypertension, coronary artery disease), and initial COVID symptoms (cough, diarrhea, fatigue, fever, flu-like symptoms, loss of appetite, shortness of breath, sputum production, vomiting). For variables with missing data (sex, BMI, and COVID hospitalization), prior to matching, missing values for sex were found in the EHR and multiple imputation using chained equations was used to fill in missing values for BMI and COVID hospitalization status (with the first of 20 imputed datasets). The greedy nearest neighbor method was used to match one PASC patient to up to three controls (1:3 matching) using the smallest within-pair difference between the propensity score logit using a caliper of 0.2 using the R package MatchIt (22). In the matched sample, covariate balance was assessed with standardized mean differences, with differences >0.1 indicating imbalance.

HRQL pre-COVID, at follow-up, and change between the two time points was summarized between PASC cases and controls using descriptive statistics and compared using linear regression via generalized estimating equations (GEEs) accounting for the match identifier. The trajectory of HRQL over time was modeled using GEEs with exchangeable correlation structure, and included time, group (case vs. control), and an interaction effect between group and time, further accounting for the match identifier and adjusting for variables with standardized differences >0.1, if any (23).

Statistical analyses were conducted using R version 4.3.1 at significance level 0.05.

## Results

There were 3,236 patients seen in the reCOVer Clinic during the study window with mean age 50.4 (SD 14.6), 70.9% female, and 79.3% white race (Table 1). Of these, 1,114 (34.4%) completed PROMIS-GH prior to their initial COVID-19 diagnosis and were included in our study. Compared to all patients seen in the reCOVer Clinic, patients included in the study were older (53.0 ± 14.0) and more likely female (74.6%). Of the patients included in the study, 24.6% were hospitalized at the time of their initial COVID-19 infection, and 5.1% required an ICU stay, which was similar to all PASC patients. Patients included in the study had more comorbidities and more initial COVID symptoms. Pre-COVID HRQL was captured a median of 15.7 (q1 = 11.1, q3 = 22.2) months before initial COVID infection and indicated worse global mental health (GMH) and physical health (GPH) scores than the general population (45.8 ± 9.4 and 43.2 ± 8.9, respectively).

**Table 1:** Patient characteristics for all patients with PASC and study sample.

**Characteristics**	**Seen in reCOVer clinic (*n* = 3,236)**	**Included in the study with pre-COVID PROMIS Global Health (*n =* 1,114)**
**Demographics**		
Age, mean ± SD	50.4 ± 14.6	53.0 ± 14.0
Female, *n* (%)	2,293 (70.9)	829 (74.6)
**Race/Ethnicity**, ***n*** **(%)**		
White	2,565 (79.3)	880 (79.0)
Black	437 (13.5)	167 (15.0)
Other/unknown	231 (7.1)	67 (6.0)
Hispanic, *n* (%)	113 (3.5)	43 (3.9)
**Comorbidities**, ***n*** **(%)**		
BMI (kg/m^2^)	31.3 ± 7.9	32.0 ± 8.0
Obesity	1,257 (50.1)	495 (54.1)
Hypertension	523 (16.2)	244 (21.9)
Asthma	359 (11.1)	160 (14.4)
Diabetes	212 (6.6)	96 (8.6)
Coronary artery disease	74 (2.3)	32 (2.9)
COPD/emphysema	53 (1.6)	21 (1.9)
Heart failure symptoms	51 (1.6)	26 (2.3)
Chronic fatigue	16 (0.5)	7 (0.6)
Dementia	5 (0.2)	3 (0.3)
**Initial COVID symptoms**, ***n*** **(%)**		
Fatigue	1,612 (50.0)	593 (53.2)
Cough	1,327 (41.2)	575 (51.6)
Shortness of breath	1,162 (36.0)	406 (36.4)
Flu-like symptoms	1,068 (33.1)	485 (43.5)
Fever	813 (25.2)	376 (33.8)
Vomiting	451 (14.0)	173 (15.5)
Loss of appetite	268 (8.3)	118 (10.6)
Sputum production	267 (8.3)	115 (10.3)
Diarrhea	234 (7.3)	98 (8.8)
**COVID outcomes**, ***n*** **(%)**		
Required hospitalization	695 (21.9)	269 (24.6)
Required ICU stay	181 (5.7)	56 (5.1)
Required intubation	74 (2.3)	19 (1.7)
**Pre-COVID HRQL, mean** ±**SD**		
PROMIS Global Mental Health	—	45.8 ± 9.4
PROMIS Global physical health	—	43.2 ± 8.9

Missing on the following for sample seen in reCOVer Clinic (n = 3,236): sex = 3; race = 3; BMI = 729; obesity = 729; initial COVID symptoms = 12; hospitalization = 69; ICU stay = 73; intubation = 75. Missing on the following for sample with pre-COVID PROMIS GH (*n* = 1,114): sex = 2; BMI = 199; obesity = 199; hospitalization = 20; ICU stay = 21; intubation=22.

**Figure 1:**
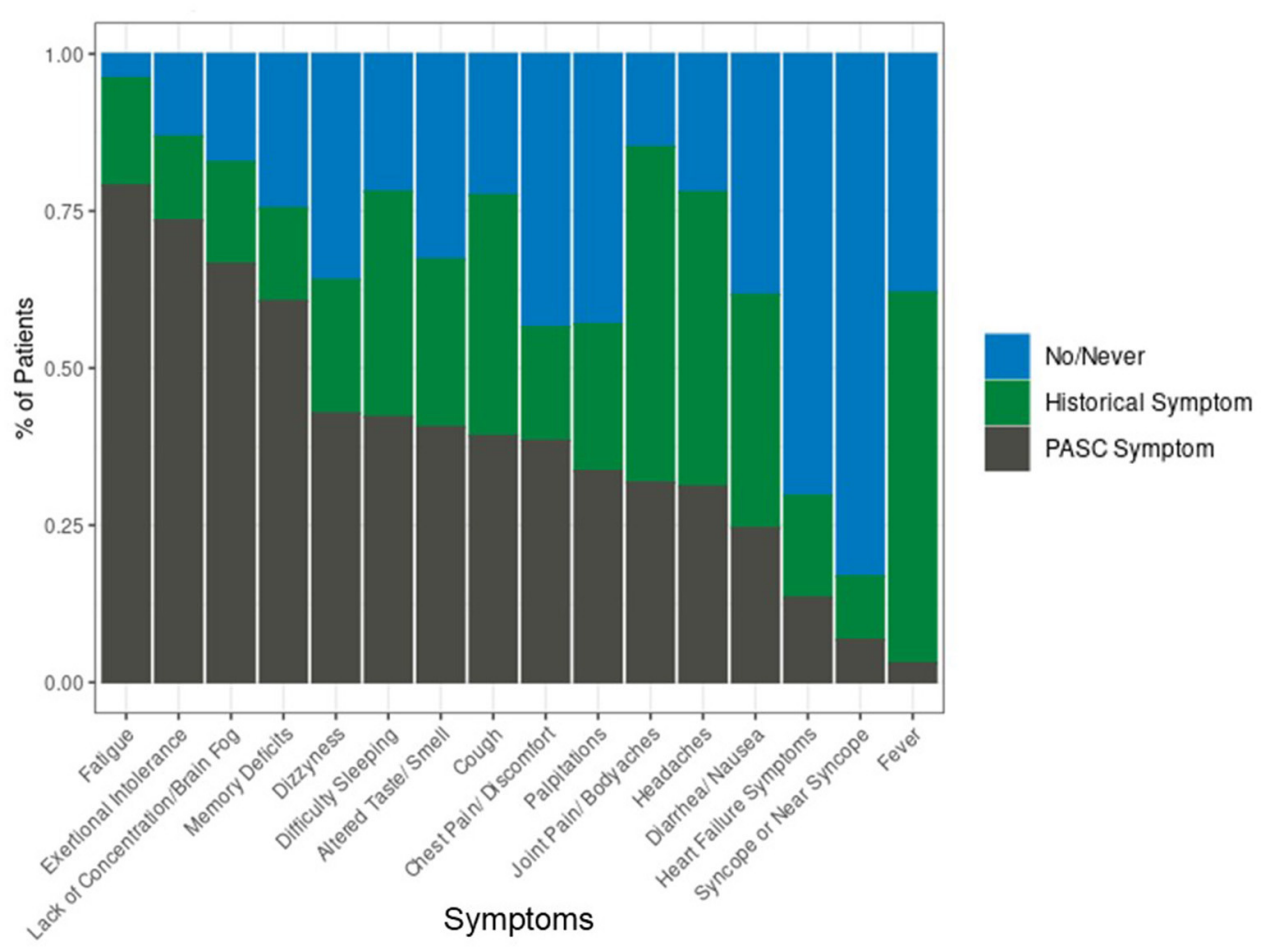
Proportion of patients with PASC experiencing symptoms, *n* = 1,114 patients.

For patients with PASC, the most prevalent COVID symptoms at the time of initial infection were fatigue (53.2%), cough (51.6%), flu-like symptoms (43.5%), and shortness of breath (36.4%). At the time of the PASC visit, PASC-related symptoms of fatigue, exertional intolerance, lack of concentration and brain fog, and memory deficits were experienced by the majority of patients (Figure 1).

After adjustment for age, sex, and initial COVID hospitalization, most PASC symptoms were significantly associated with pre-COVID mental and physical health (Table 2). Models assessed symptoms as recurred or new (i.e., PASC symptoms), present prior to COVID or resolved (i.e., Historical), compared to no/never experienced. Pre-COVID HRQL was associated with both PASC and historical symptoms, although associations were stronger for PASC symptoms than historical symptoms. PASC symptoms that were most associated with PROMIS-GMH included diarrhea/nausea [odds ratio (OR): 1.27 (95% CI: 1.16–1.39) per 5-point worsening in PROMIS-GMH] and lack of concentration/brain fog [OR: 1.25 (95% CI: 1.14–1.37)]. For PROMIS-GPH, diarrhea/nausea was also highly associated with PASC symptoms [OR: 1.32 (95% CI: 1.20–1.45) per 5-point worsening in PROMIS-GPH]. PASC-related fatigue [OR: 1.39 (95% CI: 1.15–1.68)], exertional intolerance [OR: 1.36 (95% CI: 1.22–1.52)], and joint pain/body aches [OR: 1.33 (95% CI: 1.18–1.49)] exhibited the highest associations with PROMIS-GPH.

**Table 2:** Pre-COVID PROMIS Global Mental and physical health as a predictor of PASC symptoms, *n* = 1,114.

**Symptom**	**PASC symptom (reference** = **never experienced)**		**Historical symptom (reference** = **never experienced)**	
	**aOR (95% CI)**	***p*** **-value**	**aOR (95% CI)**	***p*** **-value**
**Global mental health**				
Fatigue	1.16 (0.97–1.37)	0.10	1.12 (0.93–1.35)	0.23
Exertional intolerance	1.21 (1.10–1.34)	<0.001	1.20 (1.05–1.36)	0.007
Joint pain/body aches	1.21 (1.08–1.34)	<0.001	1.14 (1.03–1.25)	0.010
Lack of concentration/brain fog	1.25 (1.14–1.37)	<0.001	1.21 (1.08–1.36)	0.001
Cough	1.12 (1.03–1.22)	0.011	1.03 (0.94–1.12)	0.57
Headaches	1.16 (1.05–1.28)	0.003	1.05 (0.96–1.15)	0.31
Difficulty sleeping	1.12 (1.03–1.23)	0.011	1.16 (1.06–1.27)	0.001
Memory deficits	1.21 (1.11–1.31)	<0.001	1.19 (1.06–1.33)	0.003
Altered taste/smell	1.04 (0.96–1.13)	0.29	0.97 (0.89–1.06)	0.49
Dizziness	1.16 (1.08–1.25)	<0.001	1.17 (1.06–1.28)	0.001
Fever	1.13 (0.93–1.38)	0.22	1.05 (0.98–1.12)	0.17
Diarrhea/nausea	1.27 (1.16–1.39)	<0.001	1.09 (1.01–1.18)	0.034
Palpitations	1.14 (1.05–1.23)	0.002	1.15 (1.05–1.25)	0.002
Chest pain/discomfort	1.15 (1.06–1.24)	<0.001	1.06 (0.97–1.17)	0.19
Heart failure symptoms	1.05 (0.94–1.16)	0.39	1.04 (0.95–1.15)	0.42
Syncope or near syncope	1.16 (1.01–1.33)	0.036	1.09 (0.97–1.22)	0.15
**Global physical health**				
Fatigue	1.39 (1.15–1.68)	<0.001	1.29 (1.05–1.59)	0.014
Exertional intolerance	1.36 (1.22–1.52)	<0.001	1.21 (1.06–1.40)	0.007
Joint pain/body aches	1.33 (1.18–1.49)	<0.001	1.29 (1.16–1.44)	<0.001
Lack of concentration/brain fog	1.17 (1.06–1.29)	0.002	1.14 (1.00–1.29)	0.042
Cough	1.10 (1.01–1.21)	0.037	0.99 (0.91–1.09)	0.89
Headaches	1.22 (1.10–1.35)	<0.001	1.14 (1.03–1.26)	0.009
Difficulty sleeping	1.17 (1.07–1.29)	0.001	1.19 (1.08–1.31)	<0.001
Memory deficits	1.15 (1.06–1.25)	0.001	1.10 (0.97–1.23)	0.13
Altered taste/smell	1.06 (0.98–1.16)	0.16	0.97 (0.89–1.07)	0.60
Dizziness	1.21 (1.11–1.31)	<0.001	1.20 (1.09–1.32)	<0.001
Fever	1.15 (0.93–1.41)	0.20	1.05 (0.97–1.13)	0.22
Diarrhea/nausea	1.32 (1.20–1.45)	<0.001	1.15 (1.06–1.25)	<0.001
Palpitations	1.15 (1.06–1.26)	<0.001	1.14 (1.04–1.25)	0.005
Chest pain/discomfort	1.20 (1.11–1.31)	<0.001	1.10 (0.99–1.21)	0.07
Heart failure symptoms	1.17 (1.04–1.30)	0.006	1.18 (1.06–1.30)	0.002
Syncope or near syncope	1.25 (1.08–1.44)	0.003	1.10 (0.98–1.24)	0.11

Multivariable multinomial logistic regression models for pre-COVID PROMIS Global Mental and physical health as predictor of symptoms. aOR (95% CI) = adjusted odds ratio presented with confidence interval. Models adjusted for age, sex, and COVID hospitalization. Odds ratios based on a five-point worsening on PROMIS Global Health *T*-score (i.e., a clinically meaningful difference).

Of the 1,114 patients with PASC, 959 had follow-up PROMIS-GH within 1-year (median time between reCOVer Clinic visit and 1-year survey = 12.0 (q1 = 10.6, q3 = 13.0) months). For Aims 2 and 3, there were 2,513 controls matched to 940 patients with PASC (Supplemental Table 1). Characteristics were similar between the full sample of PASC patients and the 940 with pre-COVID and 1-year follow-up PROMIS-GH. The controls and PASC patients were well matched, with covariate balance obtained for all variables (standardized differences ranged from −0.05 to 0.037; Supplemental Figure 1).

Pre-COVID GMH and GPH were significantly worse for PASC patients compared to matched controls [−2.6 (SE 0.4) and −3.4 (0.3) *T*-score points, respectively; Supplemental Table 2; see Table 3 for pre-COVID and 1-year follow-up PROMIS-GH summary scores].

**Table 3:** PROMIS Global Health in patients with PASC and matched controls.

**Time Point**	**Global Mental Health** ***T*****-score**			**Global physical health** ***T*****-score**		
	**PASC**	**Controls**	***p*** **-value**	**PASC**	**Controls**	***p*** **-value**
Pre-COVID	45.94 ± 9.36	48.56 ± 9.19	<0.001	43.32 ± 8.79	46.70 ± 8.70	<0.001
1-year follow-up	43.96 ± 9.42	47.73 ± 9.24	<0.001	42.15 ± 8.73	46.91 ± 8.73	<0.001
Change	−1.98 ± 8.19^**^	−0.83 ± 7.74^**^	<0.001	−1.18 ± 7.54^**^	0.21 ± 7.40	<0.001

Values shown as mean with standard deviation;

^*^p < 0.05;

** *p* < 0.001 for change within group based on paired *t*-test; Note, meaningful change in PROMIS Global Health is considered between 2.5 and 5 *T*-score points.

**Figure 2:**
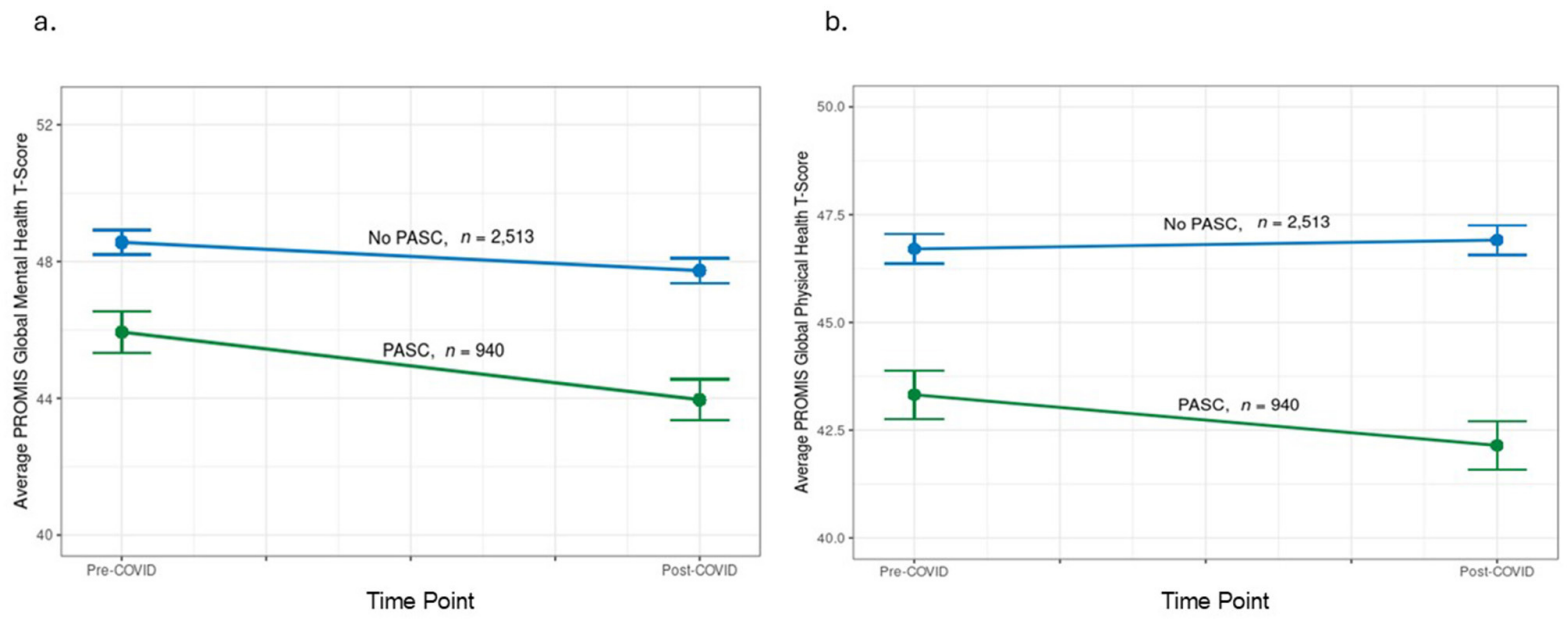
Change in PROMIS Global Health pre-COVID diagnosis to 1-year after. **(a)** Change in PROMIS Global Mental Health pre- and post- initial COVID diagnosis in patients by PASC status (PASC vs. no PASC controls); **(b)** Change in PROMIS Global physical health pre- and post- initial COVID diagnosis in patients by PASC status (PASC vs. no PASC controls). Note, meaningful change in PROMIS Global Health is considered between 2.5 and 5 *T*-score points.

One-year following COVID, the disparities were even greater for GMH and GPH in PASC patients vs. controls [−3.8 (0.4) and −4.8 (0.3), respectively]. In a GEE model, there was a significant interaction between group (cases vs. controls) and time (Supplemental Table 2). From pre-COVID to 1-year following COVID, PASC patients worsened significantly in their mental and physical health (−2.0 ± 8.2 and −1.2 ± 7.5 *T*-score points, respectively), compared to controls who worsened significantly on mental health but not physical health (−0.8 ± 7.7 and 0.2 ± 7.4 *T*-score points, respectively; Table 3, Figure 2).

## Discussion

Our study found that among patients with PASC, worse pre-COVID HRQL was associated with more PASC-related symptoms. Additionally, PASC patients had worse pre-COVID HRQL compared to matched controls. PASC patients experienced a greater decline of HRQL 1 year after their COVID-19 diagnosis compared to their pre-COVID state relative to propensity-score matched controls. However, the decline in both PASC patients and controls was slight and below the threshold for clinical significance.

Lower pre-COVID HRQL was associated with most PASC-related symptoms. Interestingly, the PASC-related symptoms that were associated with pre-COVID global mental health differed from those associated with global physical health. This aligns with findings that prior mental comorbidity is associated with an increased risk of PASC and PASC-related symptoms (14). In our study, poor concentration/brain fog and memory deficits were among the highest associations with worse pre-COVID mental health, whereas fatigue, exertional intolerance, and joint pain/body aches exhibited among the highest associations with pre-COVID physical health. When symptoms were stratified into PASC-related vs. historical, pre-COVID HRQL was associated with more PASC-related symptoms as compared to historical. This indicates the association is specific to those that are PASC-related and not between poor HRQL and a heightened sensitivity to symptoms overall.

Diarrhea/nausea was highly associated with worse pre-COVID global physical and mental health scores. This finding is noteworthy in light of the recent focus on the gut microbiome and its connection to PASC. The gut microbiota plays a role in health, mood, and illness (24, 25). Studies have shown that the composition of the microbiome is associated with COVID-19 severity, and it may contribute to persistent symptoms and inflammation following COVID illness (26). Other potential causes for the increase in diarrhea/nausea symptoms with PASC have been hypothesized as persistent viremia in the gut cells, exacerbation of irritable bowel syndrome due to post-traumatic stress of PASC, or decreased gut serotonin due to PASC (27, 28). Further study of the microbiome is necessary to identify the cause of these common symptoms.

The composition of the gut microbiome has been associated with both physical and mental HRQL (29), and a systematic review found reduced gut-microbial diversity to be associated with mental illness and chronic fatigue (30). Trials evaluating nutrition and lifestyle interventions in patients with PASC have been suggested to improve gastrointestinal issues, targeting mechanisms involving the gut microbiome (31). These types of interventions have also been shown to improve HRQL (32). Fatigue is among the most experienced symptom in PASC patients, and also a questionnaire item that comprises PROMIS global physical health (and most HRQL instruments). Interventions focused on improving fatigue and post-exertional malaise, such as a supervised activity program involving walking, stretching, or aerobic exercise (31), could be integral to improving overall HRQL.

The finding that patients with PASC had significantly lower pre-COVID HRQL compared to matched controls merits further investigation. Patients with lower HRQL may have a reduced threshold for developing PASC or heightened sensitivity to persistent symptoms, making them more likely to receive a diagnosis of PASC. The finding of worse pre-COVID HRQL in PASC patients is consistent with prior literature that has shown that prior mental comorbidity is associated with an increased risk of PASC and its associated symptoms (14).

Our study also evaluated the trajectory of HRQL from prior to COVID to 1-year following initial COVID diagnosis. Over this time, we found PASC patients worsened in both their mental and physical health [−2.0 (SD 8.2) and −1.2 (SD 7.5) *T*-score points, respectively], compared to controls who worsened in mental health but not physical health [−0.8 (SD 7.7) and 0.2 (SD 7.4) *T*-score points, respectively]. However, these differences are likely too small to be clinically meaningful, as minimal important differences are typically defined as between 2.5 and 5 *T*-score points (21). These findings suggest that even in patients with PASC, HRQL is minimally worse compared to pre-COVID levels after 1 year. Other studies concluding that HRQL is significantly affected long-term following PASC were cross-sectional and did not include baseline comparisons, which hinders interpretation (6, 18, 33). Our findings align with prior work by our group that global mental health declines (−0.85 *T*-score points) in all patients with COVID 1-year following infection compared to controls, but physical health remains stable (34). The slight decline in mental health may not be solely due to COVID: global mental health has been demonstrated to decline in all patients in our healthcare system during the pandemic compared to 1-year after (−1.21 *T*-score points) (35). While our study did not include PROMIS Global Health at the time of the reCOVer Clinic visit, prior work by our group of all patients seen in the reCOVer Clinic demonstrated global mental and physical scores of 41.5 (SD 9.2) and 39.8 (SD 6.9), which were substantially worse than those pre-COVID and 1-year post-PASC visit (36). This suggests that patients with PASC likely experience a reduction in HRQL before improving to near pre-COVID levels at 1-year.

Our study has important clinical implications. The risk of PASC remains substantial [3.5 events per 100 vaccinated persons during the omicron-dominant period (37)] and its underlying mechanisms remain largely unknown. Our study suggests poor pre-COVID HRQL may predispose patients to PASC. In addition to contributing to the understanding of PASC mechanisms, this study offers insights for patient management. The association between poor pre-COVID HRQL and more PASC symptoms suggests that patients with low premorbid HRQL may benefit from closer monitoring for the development of post-COVID conditions. For patients with COVID-19, particularly those diagnosed with PASC, providers can offer reassurance that HRQL generally approaches pre-COVID levels within 1 year of diagnosis.

Our study includes a large sample of patients with PASC who had HRQL prior to their COVID-19 infection in our health system. It also included a propensity-score matched control group to aid interpretation of results. Despite these strengths, our study also has limitations. A primary limitation is the possibility that some patients in the control group may have had undiagnosed or mild PASC or were not referred to the PASC clinic. However, if this were the case, as the PASC clinic was internally publicized and utilized, it is likely that these patients experienced less severe PASC symptoms. The study was comprised of patients from a specialized PASC clinic, which may include patients with more severe symptoms and limit generalizable to the broader PASC population, however the percentage of patients experiencing PASC-related symptoms is similar to the reported literature. Additionally, the study period spanned multiple phases of the COVID-pandemic which included multiple variants. Testing for specific variants was not conducted, and symptoms or severity may differ based on variant, however the broad findings should remain consistent across all variants of COVID-19. Patients included in the study with completed PROMIS Global Health were slightly older and had more comorbidities compared to patients without completed PROMIS scores, therefore, our findings may not be generalizable to all patients with PASC and could be slightly more severe than those of all patients with PASC. Lastly, while PROMIS Global Health provides a general assessment of overall health-related quality of life, it may not be sensitive to changes in some COVID-related symptoms, such as shortness of breath or nausea/vomiting.

In conclusion, our study found an association between pre-COVID HRQL and diagnosis of PASC, with worse pre-COVID HRQL predicting more PASC symptoms. HRQL at 1 year was similar to pre-COVID HRQL for both patients with and without PASC providing reassuring information that patients with PASC will likely improve by 1-year post-COVID.

## Data Availability

Data will be shared upon reasonable request. Requests to access these datasets should be directed to Brittany Lapin, lapinb@ccf.org.
