# Supplementary material for: Pre-COVID health-related quality of life predicts symptoms and outcomes for patients with long COVID
Source: Front Public Health. 2025 Jul 11;13:1581288. doi: 10.3389/fpubh.2025.1581288 (PMC12289640; doi:10.3389/fpubh.2025.1581288)
Supplement: Supplementary file 1 [file Table_1.docx]

**Supplemental Table 1. Characteristics of Propensity Score-Matched PASC Patients and Controls**

| **Characteristic** | **Total**  **(*N* = 3,453)** | **PASC**  **(*N = 940)*** | **Controls**  **(*N = 2,513*)** | **Standardized difference** |
| --- | --- | --- | --- | --- |
| Age, mean + SD | 52.9 + 15.4 | 52.6 + 13.7 | 52.4 + 16.0 | 0.015 |
| Female, n (%) | 2,545 (73.7) | 704 (74.9) | 1859 (74.0) | 0.022 |
| Race, n (%) |  |  |  |  |
| White | 2,802 (81.1) | 756 (80.4) | 2003 (79.7) | 0.018 |
| Black/ African American | 480 (13.9) | 133 (14.1) | 370 (14.7) | -0.016 |
| Other/ Unknown | 171 (5.0) | 51 (5.4) | 140 (5.6) | -0.006 |
| BMI, mean + SD | 31.7 + 8.4 | 32.0 + 8.0 | 32.0 + 8.9 | 0.001 |
| Comorbidities, n (%) |  |  |  |  |
| Asthma | 541 (15.7) | 146 (15.5) | 379 (15.1) | 0.013 |
| Diabetes | 323 (9.4) | 82 (8.7) | 233 (9.3) | -0.020 |
| Hypertension | 900 (26.1) | 219 (23.3) | 621 (24.7) | -0.034 |
| Coronary Artery Disease | 129 (3.7) | 27 (2.9) | 93 (3.7) | -0.050 |
| Hospitalized for COVID, n (%) | 706 (20.4) | 226 (24.0) | 607 (24.2) | -0.003 |
| Initial COVID Symptoms, n (%) |  |  |  |  |
| Cough | 1,817 (52.6) | 489 (52.0) | 1261 (50.2) | 0.037 |
| Diarrhea | 262 (7.6) | 80 (8.5) | 207 (8.2) | 0.010 |
| Fatigue | 1855 (53.7) | 507 (53.9) | 1335 (53.1) | 0.016 |
| Fever | 1157 (33.5) | 317 (33.7) | 806 (32.1) | 0.035 |
| Flu-like Symptoms | 1511 (43.8) | 414 (44.0) | 1065 (42.4) | 0.034 |
| Loss of Appetite | 307 (8.9) | 93 (9.9) | 252 (10.0) | -0.004 |
| Shortness of Breath | 1244 (36.0) | 352 (37.4) | 950 (37.8) | -0.008 |
| Sputum Production | 340 (9.8) | 97 (10.3) | 236 (9.4) | 0.031 |
| Vomiting | 519 (15.0) | 144 (15.3) | 398 (15.8) | -0.014 |
| Months between Pre-COVID and 1-Year Follow-Up PROMIS-GH, median (q1, q3) | 40.8 (34.9, 46.9) | 37.9 (31.7, 44.4) | 41.9 (36.1, 47.5) | n/a |
