# Supplementary material for: Pre-COVID health-related quality of life predicts symptoms and outcomes for patients with long COVID
Source: Front Public Health. 2025 Jul 11;13:1581288. doi: 10.3389/fpubh.2025.1581288 (PMC12289640; doi:10.3389/fpubh.2025.1581288)
Supplement: Supplementary file 2 [file Table_2.docx]

**Supplemental Table 2. Difference in PROMIS Global Health in patients with PASC versus propensity-score matched controls**

| **Differences** | **Global Mental Health T-score** | | **Global Physical Health T-score** | |
| --- | --- | --- | --- | --- |
|  | **Mean (SE)** | **p-value** | **Mean (SE)** | **p-value** |
| Pre-COVID differences between PASC and Controls | -2.62 (0.36) | <0.001 | -3.39 (0.34) | <0.001 |
| Post-COVID differences between PASC and Controls | -3.78 (0.36) | <0.001 | -4.77 (0.33) | <0.001 |
| Change in PASC patients compared to Controls | Time:  -0.83 (0.16)  Interaction: -1.15 (0.30) | <0.001 | Time: 0.21 (0.15)  Interaction: -1.38 (0.29) | <0.001 |

SE = standard error; Negative estimates indicate worse health-related quality of life for patients with PASC compared to matched controls based on GEE models. Interaction effects graphed in Figures 2-3.
