# Supplementary material for: Pre-COVID health-related quality of life predicts symptoms and outcomes for patients with long COVID
Source: Front Public Health. 2025 Jul 11;13:1581288. doi: 10.3389/fpubh.2025.1581288 (PMC12289640; doi:10.3389/fpubh.2025.1581288)
Supplement: Supplementary file 3 [file Image_1.pdf]

Supplemental Figure 1. Covariate balance between patients with PASC and matched controls

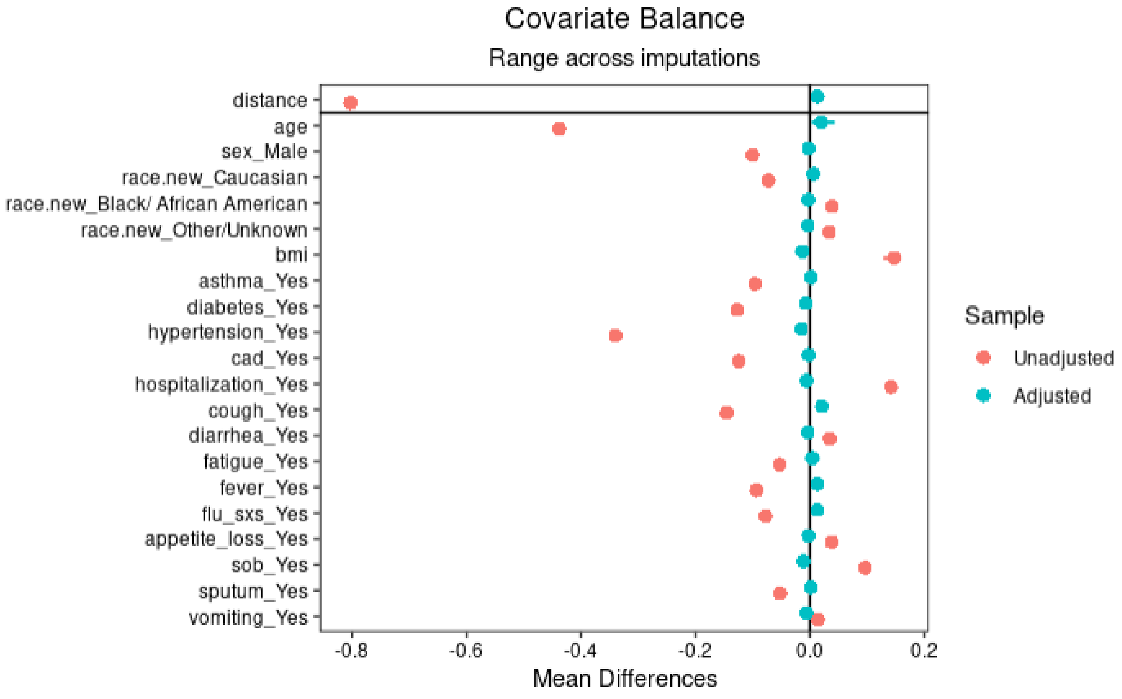

Note, balance was defined as mean differences within -0.1 and 0.1.
